# Acute Lymphoblastic Leukemia and Associated HLA-A, B, DRB1, and DQB1 Molecules: A Moroccan Pediatric Case–Control Study

**DOI:** 10.3390/ijms26115295

**Published:** 2025-05-30

**Authors:** Khalid Laaziri, Abdelmajid Zyad, El Mehdi Laaziaf, Jamila El Houdzi, Fatimaezzahra Elhanafi, Ikram Brahim, Nadia Lakhouaja, Raja Hazime, Mounia Ammara, Abdellah Naya, Brahim Admou

**Affiliations:** 1 Immunology and Biodiversity Laboratory, Ain Chock Faculty, Science Hassan II University, Casablanca 20000, Morocco; mehdilaaziaf1@gmail.com (E.M.L.); ammara.mounia18@gmail.com (M.A.); anaya@mabiotech.com (A.N.); 2 Laboratory of Immunology and HLA, Center of Clinical Research, Mohammed VI University Hospital, Marrakech 40080, Moroccobr.admou@uca.ac.ma (B.A.); 3 Laboratory of Agro-Industrial and Medical Biotechnology, Experimental Oncology and Natural Substances Team, Cellular and Molecular Immunopharmacology, Faculty of Sciences and Technology, Sultan Moulay Slimane University, Beni Mellal 23000, Morocco; a.zyad@usms.ma; 4 Department of Pediatric Hematology and Oncology, Mohammed VI University Hospital, Marrakech 40080, Morocco; 5 Bioscience Research Laboratory, Faculty of Medicine and Pharmacy, Cadi Ayyad University, Marrakech 40080, Morocco

**Keywords:** acute lymphoid leukemia, children, HLA-A, HLA-B, DRB1, DQB1, Morocco

## Abstract

Leukemia constitutes approximately one-third of all pediatric cancers, with acute lymphoblastic leukemia (ALL) comprising roughly 80% of pediatric leukemia instances. This study sought to ascertain the prevalence of HLA A, B, DR, and DQ allele groups linked with pediatric acute leukemia. We recruited 70 Moroccan children diagnosed with acute lymphoblastic leukemia (ALL), 39 of whom had BCP-ALL and were eligible for hematopoietic stem cell transplantation, compared to a control group of 136 healthy children. Patients and controls were subjected to HLA class I and II typing, utilizing either sequence-specific primers (SSPs) or sequence-specific oligonucleotides (SSOs) in polymerase chain reaction-based techniques. The findings indicated significantly elevated frequencies of HLA-A*68 and B*14 in pediatric patients with ALL relative to the control group (*p* = 0.001 and *p* = 0.02, respectively). The frequencies of HLA-DRB1*01 and DQB1*05 allele groups were considerably elevated in children with ALL and BCP-ALL compared to the controls (*p* < 0.01 for both). The findings of our study indicate that HLA-A*68, -B*14, -DRB1*01, and DQB1*05 may serve as potential predisposing immunogenetic variables for the development of juvenile acute lymphoblastic leukemia (ALL). Nonetheless, additional research including a bigger sample considering other regions of Morocco would be beneficial to more accurately delineate the association between the HLA system and ALL.

## 1. Introduction

Leukemia constitutes approximately one-third of all pediatric cancers, predominantly represented by acute lymphoblastic leukemia (ALL) [1], which accounts for 80% of pediatric leukemia cases [2]. About 70% of childhood ALL is classified as B-cell precursor acute lymphoblastic leukemia (BCP-ALL), typically affecting children aged 3 to 5 [3]. ALL arises from hematopoietic stem and progenitor cells that fail to differentiate, self-renew, and undergo apoptosis [2]. The International Agency for Research on Cancer (IARC) estimates the overall incidence of acute lymphoblastic leukemia (ALL) at 1.7 per 100,000 [4]. In Morocco, the prevalence and incidence of leukemia are estimated at 2.5 and 3.8 per 100,000, respectively, with a mortality rate of 2.6 [5].

Genes located in the human leukocyte antigen (HLA) regions, which encode class I (A, B, C) and class II (DR, DQ, DP) HLA molecules, have been linked to numerous diseases, including autoimmunity, infections, and cancer, with a particular emphasis on HLA-DRB1 alleles. Numerous studies conducted over recent decades have demonstrated a correlation between specific HLA genes and childhood acute lymphoblastic leukemia (ALL), revealing both predisposing and protective HLA alleles that vary across different populations [6]. The correlation between HLA genes and pediatric acute lymphoblastic leukemia (ALL) has been documented in various countries, including Lebanon [7], the United States [8], Mexico [9], China [10], Turkey [11], India [12], and Romania [13].

The association between HLA genes and susceptibility to ALL is still debated, necessitating additional research involving patient cohorts from diverse backgrounds, including our own context.

This study aimed to assess the distribution and frequency of HLA A, B, DR, and DQ loci in acute leukemia among a Moroccan pediatric population, with the objective of identifying potential predisposing or protective HLA alleles.

## 2. Results

### 2.1. Patient Characteristics

The mean age of all patients was 9.25 ± 8.75 years (range: 0.5 to 18 years), compared to 10 ± 8 years in the control group (range: 2 to 18 years). The study revealed a male predominance of 64.28%, resulting in a male-to-female sex ratio of 1.69, compared to 1.26 in the control group all patients’ characteristics as shown in Table 1.

**Table 1:** Patient demographic characteristics.

Characteristics	Acute Lymphoblastic Leukemia Patients *n* (%)
Total patient	70
Median age, years, range	9.25 (0.5–18)
Type of acute lymphoid leukemia	
ALL B	39 (55.71)
ALL T	23 (32.85)
Unclassified	8 (11.42)
Gender	
Female	26 (62.85)
Male	44 (37.14)
ABO groups	
A+	17 (24.28)
A−	0 (0)
B+	14 (20)
B−	0 (0)
AB+	3 (4.28)
AB−	0 (0)
O+	25 (35.71)
O-	3 (4.28)
Unclassified	8 (11.42)
Children’s race	
White	61 (87.14)
Black	9 (12.85)

### 2.2. Distribution of HLA Loci and Allele Groups in Patients with ALL and Controls

**Figure 1:**
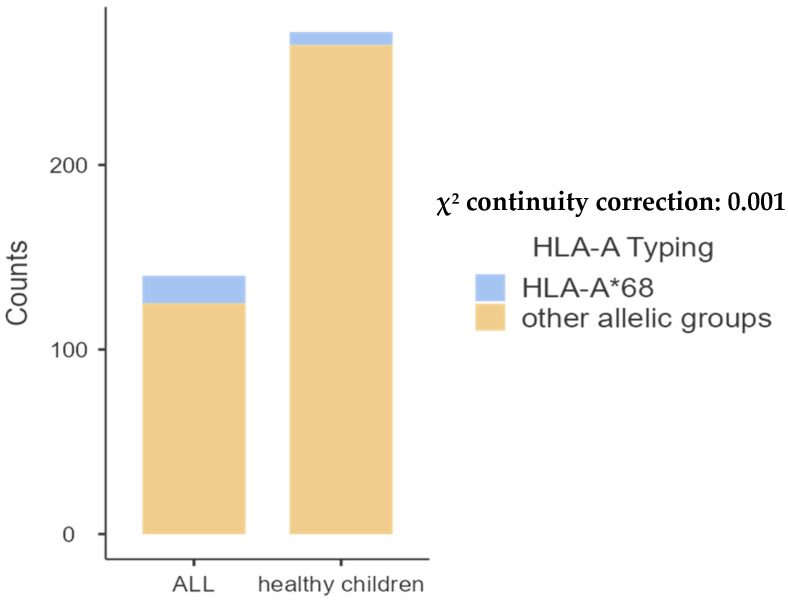
Comparative frequency of HLA-A*68 between children with ALL and controls.

**Figure 2:**
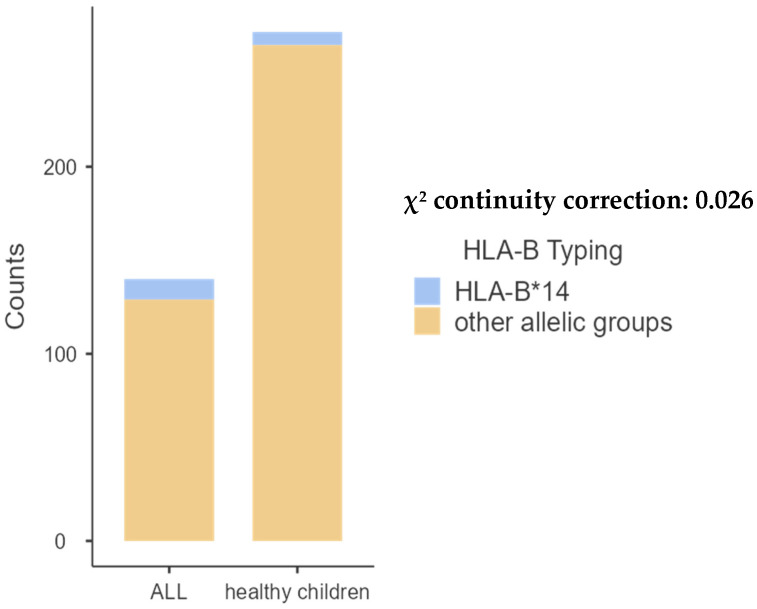
Comparative frequency of HLA-B*14 between children with ALL and controls.

**Figure 3:**
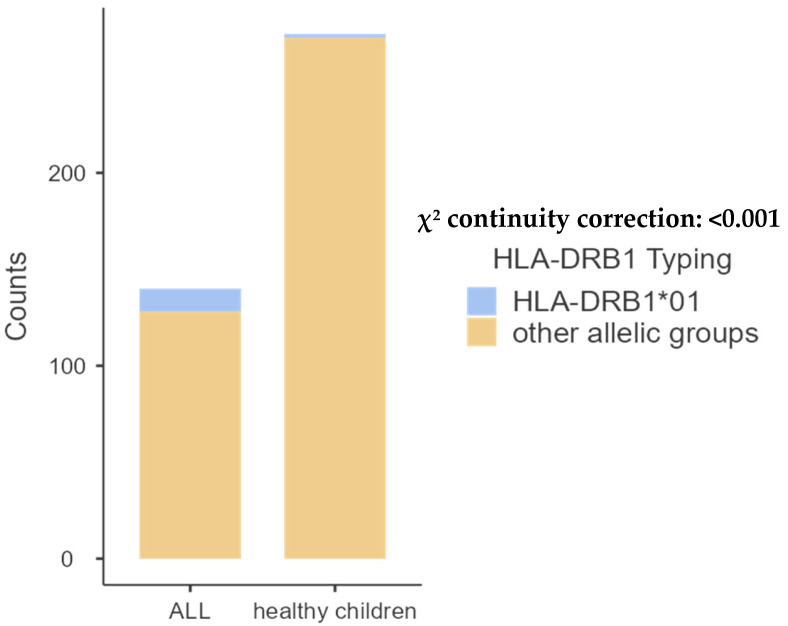
Comparative frequency of HLA-DRB1*01 between patients with ALL and controls.

**Figure 4:**
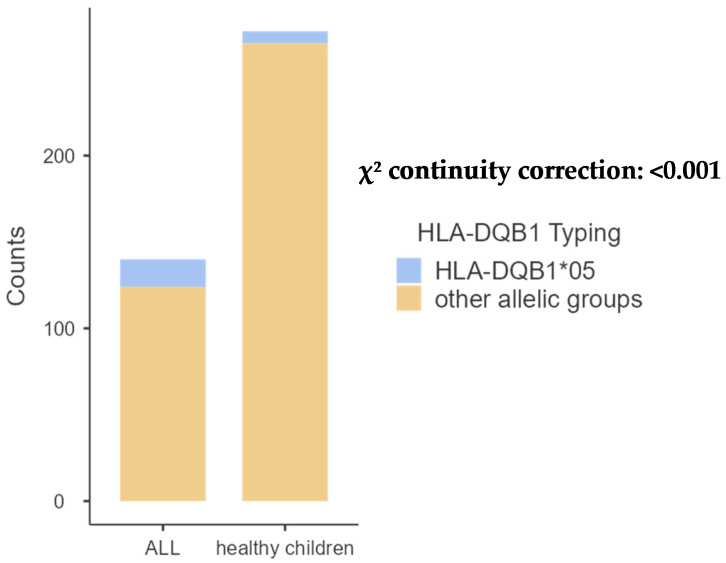
Comparative frequency of HLA-DQB1*01 between patients with ALL and controls.

The comparison of pediatric patients with the control group revealed significant differences in the frequencies of A68 (*p* = 0.001) and B14 (*p* = 0.02). Additionally, a significant difference was observed in the frequency of DRB1*01 (*p* < 0.001) and DQB1*05 (*p* < 0.01), as shown in Table 2, Table 3, Table 4 and Table 5 and Figure 1, Figure 2, Figure 3 and Figure 4 bellow. The examination of allelic group frequencies by gender revealed no statistically significant differences.

- HLA-A allele groups.

Table 2 illustrates the distribution of allelic groups within the study population. Among the 20 identified HLA-A allelic groups, HLA-A*02 was the most common in children with ALL, representing 18.57% of cases compared to 23.89% in the control group.

**Table 2:** Comparative frequencies of HLA-A allele groups between patients with ALL and controls.

HLA-A	Frequency of Allele Groups in Patients with ALL 2*n* = 140; *n* (%)	Frequency of Allele Groups in Control Group 2*n* = 272; *n* (%)	*p*-Value
A*02	26 (18.57%)	65 (23.89%)	0.26
A*30	7 (5.00%)	24 (8.82%)	0.23
A*68	15 (10.71%)	7 (2.57%)	**0.001**
A*01	12 (8.57%)	38 (13.97%)	0.15
A*03	17 (12.14%)	26 (9.55%)	0.52
A*24	9 (6.42%)	15 (5.51%)	0.87
A*33	5 (3.57%)	18 (6.61%)	0.29
A*29	5 (3.57%)	11 (4.04%)	0.97
A*11	8 (5.71%)	6 (2.20%)	0.11
A*32	7 (5.00%)	13 (4.77%)	0.88
A*23	13 (9.28%)	24 (8.82%)	0.97
A*26	4 (1.92%)	5 (1.83%)	0.75
A*31	2 (2.85%)	5 (1.83%)	0.92
A*34	2 (2.85%)	4 (1.47%)	0.68
A*36	1 (0.71%)	0 (0.00%)	0.73
A*25	1 (0.71%)	1 (0.36%)	0.78
A*28	2 (2.85%)	0 (0.00%)	0.21
A*66	2 (2.85%)	6 (2.20%)	0.86
A*74	1 (0.71%)	0 (0.00%)	0.75
A*80	1 (0.71%)	4 (1.47%)	0.85

- HLA-B allele groups.

Among the 31 HLA-B genes analyzed, HLA-B*44 was the most prevalent, occurring in 10.71% of patients and 10.66% of controls.

**Table 3:** Comparative frequencies of HLA-B allele groups between patients with ALL and controls.

HLA-B	Frequency of Allele Groups in Patients with ALL 2*n* = 140; *n* (%)	Frequency of Allele Groups in Control Group 2*n* = 272; *n* (%)	*p*-Value
B*15	3 (2.14%)	10 (3.67%)	0.58
B*13	0 (0.00%)	5 (1.83%)	0.25
B*07	11 (7.85%)	12 (4.41%)	0.22
B*14	11 (7.85%)	7 (2.57%)	**0.02**
B*44	15 (10.71%)	29 (10.66%)	0.87
B*50	12 (8.57%)	17 (6.25%)	0.50
B*35	4 (2.85%)	15 (5.51%)	0.33
B*51	12 (8.57%)	12 (4.41%)	0.13
B*08	7 (5%)	12 (4.41%)	0.98
B*18	7 (5%)	10 (3.67%)	0.70
B*49	9 (6.42%)	16 (5.88%)	**<0.001**
B*41	3 (2.14%)	11 (4.04%)	0.47
B*27	6 (4.28%)	9 (3.30%)	0.82
B*42	3 (2.14%)	6 (2.20%)	0.75
B*53	4 (2.85%)	11 (4.04%)	0.74
B*45	7 (5%)	20 (7.35%)	0.48
B*38	5 (3.57%)	8 (2.94%)	0.96
B*40	0 (0%)	10 (3.67%)	0.05
B*78	1 (0.71%)	2 (0.73%)	0.55
B*57	2 (1.42%)	17 (6.25%)	**0.04**
B*52	2 (1.42%)	5 (1.83%)	0.92
B*17	1 (0.71%)	2 (0.73%)	0.55
B*39	3 (2.14%)	7 (2.57%)	0.94
B*58	5 (3.57%)	11 (4.04%)	0.97
B*55	0 (0.0%)	2 (0.73%)	0.78
B*37	2 (1.42%)	0 (1.04%)	0.21
B*72	3 (2.14%)	3 (1.10%)	0.68
B*63	2 (1.42%)	0 (0.0%)	0.21
B*62	0 (0.0%)	2 (0.73%)	0.78
B*55	0 (0.0%)	2 (0.73%)	0.78
B*25	0 (0.0%)	1 (0.36%)	0.73

- HLA-DRB1 allele groups.

Among class II HLA molecules, DRB1*03 was the predominant allele in patients, accounting for 24.00%, while the control group exhibited 12 allele groupings, with HLA-DRB1*04 being the most prevalent at 15.26%.

**Table 4:** Comparative frequencies of HLA-DRB1 allele groups between patients with ALL and controls.

HLA-DRB1	Frequency of Allele Groups in Patients with ALL 2*n* = 110; *n* (%)	Frequency of Allele Groups in Control Group 2*n* = 190; *n* (%)	*p*-Value
DRB1*03	24 (24.00%)	33 (17.36%)	0.42
DRB1*13	17 (15.45%)	38 (20%)	0.40
DRB1*04	14 (12.72%)	29 (15.26%)	0.66
DRB1*15	10 (9.09%)	28 (14.73%)	0.21
DRB1*11	11 (10%)	9 (4.73%)	0.12
DRB1*07	17 (15.45%)	33 (17.36%)	0.78
DRB1*01	12 (10.90%)	2 (1.05%)	**0.0002**
DRB1*08	2 (1.81%)	8 (4.21%)	0.43
DRB1*10	1 (0.90%)	1 (0.52%)	0.73
DRB1*14	2 (1.81%)	4 (2.10%)	0.79
DRB1*09	0 (0.00%)	4 (2.10%)	0.31
DRB1*16	0 (0.00%)	1 (0.52%)	0.78

- HLA-DQB1 allele groups.

Our study identified five allele categories for HLA-DQB1, with HLA-DQB1*02 exhibiting the highest frequency in the ALL group at 34.54%, compared to 36.31% in the control group.

**Table 5:** Comparative frequencies of HLA-DQB1 allele groups between patients with ALL and controls.

HLA-DQB1	Frequency of Allele Groups in Patients with ALL 2*n* = 110; *n* (%)	Frequency of Allele Groups in Control Group 2*n* = 190; *n* (%)	*p*-Value
DQB1*02	38 (34.54%)	69 (36.31%)	0.85
DQB1*03	25 (22.72%)	46 (24.21%)	0.88
DQB1*04	5 (4.54%)	15 (7.89%)	0.37
DQB1*05	16 (14.54%)	7 (3.68%)	**0.001**
DQB1*06	26 (23.63%)	53(27.89%)	0.50

### 2.3. Distribution of HLA Loci and Allele Groups in Patients with BCP-ALL and Controls

**Figure 5:**
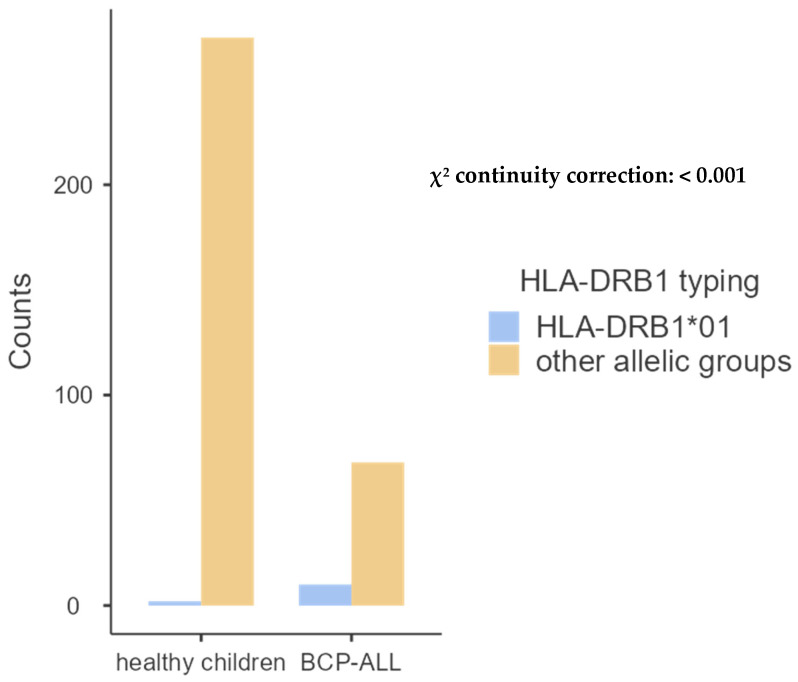
Comparative frequency of HLA-DRB1*01 between patients with BCP-ALL and controls.

**Figure 6:**
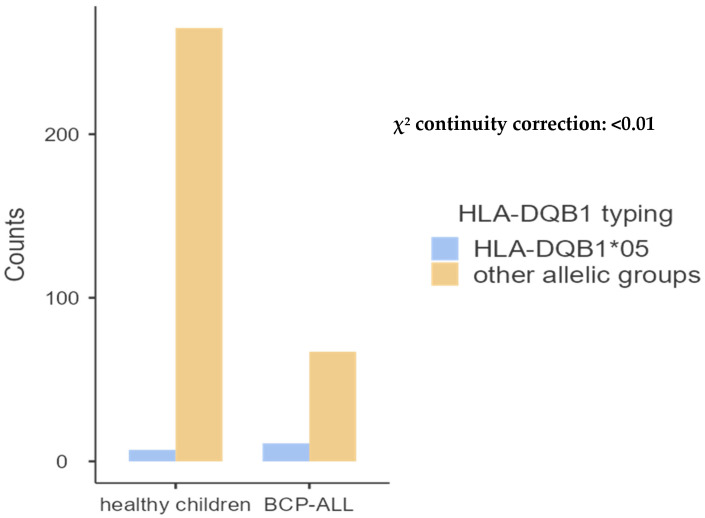
Comparative frequency of HLA-DQB1*01 between patients with BCP-ALL and controls.

We also analyzed the correlation between HLA molecules in 39 children with BCP-ALL and the same 136 healthy juveniles studied above, discovering a relationship solely with HLA class II molecules—DRB1*01 and DQB1*05—as demonstrated in Table 6, Table 7, Table 8 and Table 9 and Figure 5 and Figure 6 below.

- HLA-A allele groups.

As shown in Table 5, the distribution of allelic groups in the study population revealed that of the 16 HLA-A allelic groups identified, HLA-A*02 was the most prevalent in children with BCP-ALL, comprising 16.66% of cases compared to 23.89% in the controls; nevertheless, no correlation was detected as shown in Table 6 bellow.

**Table 6:** Comparative frequencies of HLA-A allele groups between patients with BCP-ALL and controls.

HLA-A	Frequency of Allele Groups in Patients with BCP-ALL 2*n* = 78; *n* (%)	Frequency of Allele Groups in Control Group 2*n* = 272; *n* (%)	*p*-Value
A*02	13 (16.66%)	65 (23.89%)	0.23
A*30	4 (5.12%)	24 (8.82%)	0.41
A*68	3 (3.84%)	7 (2.57%)	0.83
A*01	6 (7.69%)	38 (13.97%)	0.20
A*03	11 (14.10%)	26 (9.55%)	0.34
A*24	6 (7.69%)	15 (5.51%)	0.65
A*33	4 (5.12%)	18 (6.61%)	0.83
A*29	3 (3.84%)	11 (4.04%)	0.80
A*11	5 (6.41%)	6 (2.20%)	0.21
A*32	6 (7.69%)	13 (4.77%)	0.13
A*23	9 (11.53%)	24 (8.82%)	0.61
A*26	3 (3.84%)	5 (1.83%)	0.51
A*34	1 (1.28%)	4 (1.47%)	0.67
A*25	1 (1.28%)	1 (0.36%)	0.92
A*28	2 (2.56%)	0 (0.00%)	0.50
A*66	1 (1.28%)	6 (2.20%)	0.95

- HLA-B allele groups.

Regarding the 30 HLA-B genes observed, HLA-B*44 was the most frequent in patients, with 12.82% in patients and 10.66% in controls, but no association was found as shown in Table 7 bellow.

**Table 7:** Comparative frequencies of HLA-B allele groups between patients with BCP-ALL and controls.

HLA-B	Frequency of Allele Groups in Patients with BCP-ALL 2*n* = 78; *n* (%)	Frequency of Allele Groups in Control Group 2*n* = 272; *n* (%)	*p*-Value
B*15	1 (1.28%)	10 (3.67%)	0.50
B*13	0 (0.00%)	5 (1.83%)	0.50
B*07	7 (8.97%)	12 (4.41%)	0.18
B*14	5 (6.41%)	7 (2.57%)	0.88
B*44	10 (12.82%)	29 (10.66%)	0.74
B*50	7 (8.97%)	17 (6.25%)	0.55
B*35	3 (3.84%)	15 (5.51%)	0.76
B*51	9 (11.53%)	12 (4.41%)	0.03
B*08	5 (6.41%)	12 (4.41%)	0.67
B*18	3 (3.84%)	10 (3.67%)	0.78
B*49	4 (5.12%)	16 (5.88%)	0.98
B*41	2 (2.56%)	11 (4.04%)	0.78
B*27	2 (2.56%)	9 (3.30%)	0.97
B*42	1 (1.82%)	6 (2.20%)	0.95
B*53	2 (2.56%)	11 (4.04%)	0.78
B*45	5 (6.41%)	20 (7.35%)	0.97
B*38	3 (3.84%)	8 (2.94%)	0.97
B*40	0 (0.00%)	10 (3.67%)	0.18
B*78	1 (1.28%)	2 (0.73%)	0.81
B*57	1 (1.28%)	17 (6.25%)	0.14
B*52	2 (2.56%)	5 (1.83%)	0.95
B*17	0 (0.00%)	2 (0.73%)	0.92
B*39	0 (0.00%)	7 (2.57%)	0.33
B*58	1 (1.28%)	11 (4.04%)	0.40
B*37	1 (1.28%)	0 (0.00%)	0.50
B*72	2 (2.56%)	3 (1.10%)	0.67
B*63	1 (1.28%)	0 (0.0%)	0.50
B*62	0 (0.00%)	2 (0.73%)	0.92
B*55	0 (0.00%)	2 (0.73%)	0.92
B*25	0 (0.00%)	1 (0.36%)	0.50

- HLA-DRB1 allele groups.

For class II HLA molecules, DRB1*03 was the most frequent among the 9 allele groups found in patients, with 23.61%, whereas in the control group, 12 allele groups were found and HLA-DRB1*04 was the most common, with 15.26%. We found that the frequency of DRB1*01 was higher in children with BCP-ALL than in the control group (*p* < 0.001), as shown in Table 8 and Figure 5.

**Table 8:** Comparative frequencies of HLA-DRB1 allele groups between patients with BCP-ALL and controls.

HLA-DRB1	Frequency of Allele Groups in Patients with BCP-ALL 2*n* = 72; *n* (%)	Frequency of Allele Groups in Control Group 2*n* = 190; *n* (%)	*p*-Value
DRB1*03	17 (23.61%)	33 (17.36%)	0.33
DRB1*13	11 (15.27%)	38 (20%)	0.48
DRB1*04	10 (13.88%)	29 (15.26%)	0.93
DRB1*15	7 (9.72%)	28 (14.73%)	0.38
DRB1*11	6 (8.33%)	9 (4.73%)	0.41
DRB1*07	9 (12.50%)	33 (17.36%)	0.44
DRB1*01	10 (13.88%)	2 (1.05%)	**0.00004**
DRB1*08	1 (1.38%)	8 (4.21%)	0.45
DRB1*14	1 (1.38%)	4 (2.10%)	0.89
DRB1*09	0 (0.00%)	4 (2.10%)	0.49
DRB1*16	0 (0.00%)	1 (0.52%)	0.61

- HLA-DQB1 allele groups.

For HLA-DQB1, the study revealed the presence of five allele groups, and HLA-DQB1*02 was the highest in the ALL group with a frequency of 33.33% versus 36.31% in the control group. We discovered that children with BCP-ALL had a greater frequency of DQB1*05 than the control group (*p* < 0.01), as indicated in Table 9 and Figure 6.

**Table 9:** Comparative frequencies of HLA-DQB1 allele groups between patients with BCP-ALL and controls.

HLA-DQB1	Frequency of Allele Groups in Patients with ALL B 2*n* = 72; *n* (%)	Frequency of Allele Groups in Control Group 2*n* = 190; *n* (%)	*p*-Value
DQB1*02	24 (33.33%)	69 (36.31%)	0.75
DQB1*03	17 (23.61%)	46 (24.21%)	0.95
DQB1*04	2 (2.77%)	15 (7.89%)	0.22
DQB1*05	11 (15.27%)	7 (3.68%)	**0.002**
DQB1*06	18 (25.00%)	53 (27.89%)	0.75

### 2.4. Distribution of HLA Loci and Allele Groups in Patients with TCP-ALL and Controls

Since the population of children with T-cell precursor acute lymphoblastic leukemia (TCP-ALL) was limited to 23 pediatric patients, which is not statistically significant, the study of the association of HLA-A, -B, -DRB1, and DQB1 molecules with TCP-ALL was not carried out.

## 3. Discussion

Leukemia is affected by both environmental and genetic risk factors. In adults, cancer development is significantly impacted by lifestyle factors such as body weight, diet, physical activity, and tobacco use; however, these factors generally require years to exert their influence and are not regarded as primary contributors to childhood cancers, including leukemia [14]. While certain studies have indicated a correlation between passive smoking and ALL, the findings remain contentious [15]. Environmental risk factors for leukemia encompass exposure to hazardous air pollutants (HAPs), radiation, and specific chemicals such as household solvents and pesticides. Moreover, Wiskott–Aldrich syndrome and other hereditary immune system illnesses elevate the chance of leukemia, particularly due to genetic factors; for instance, conditions such as Down syndrome and Li–Fraumeni syndrome are recognized to augment the likelihood of leukemia [14].

Numerous prenatal chromosomal abnormalities have been correlated with leukemia, with hyperdiploidy being the predominant chromosomal anomaly associated with leukemogenesis [15]. Additionally, siblings of children with leukemia exhibit a marginally elevated risk of developing the disease, while the overall risk remains minimal. The risk is markedly elevated in identical twins; if one twin is diagnosed with leukemia during the first year of life, the other twin has an approximately 20% probability of also developing the disease [14].

The current study indicates a heightened prevalence of particular HLA alleles in juvenile acute lymphoblastic leukemia (ALL) relative to the control group. We identified that for HLA class I, the allele A*68 at locus A (*p* = 0.001) and the allele B*14 at locus B (*p* = 0.02) are significant. For HLA class II, the alleles DRB1*01 (*p* < 0.001) and DQB1*05 (*p* < 0.01) are also significant. In this context, all four alleles—A*68, HLA-B*40, and B*53—were identified in a U.S. population, suggesting that these alleles confer a heightened risk of juvenile leukemia [9]. Additionally, the HLA-A*30, HLA-A*68, and HLA-B*40 alleles were identified in Lebanese [7], and Mexican populations, respectively [9]. A prior study conducted in Morocco on the general populace revealed a notable prevalence of specific alleles, namely HLA-B*44 (12.7%; *p* = 0.02) and HLA-DRB1*13 (11.8%; *p* = 0.04), alongside a diminished allelic frequency of HLA-DRB1*01 (4.5%; *p* = 0.05). The data indicate a statistically significant correlation between these HLA alleles and various forms of leukemia in Moroccan patients, suggesting that HLA-B*44, HLA-DRB1*01, and HLA-DRB1*13 may either predispose persons to or confer protection against leukemia. Nevertheless, further extensive research is required to validate these correlations [16].

Our investigation demonstrated that the frequencies of the HLA-DRB1*01 allele in our patients were considerably elevated compared to the control group (*p* = 0.03). A relationship with the DRB1*01 allele has been recognized in the Turkish population, indicating that this allele constitutes a risk factor for ALL [10]. In our investigation, the HLA-DQB1*05 gene was identified as a genetic risk factor specifically for juvenile acute lymphoblastic leukemia (ALL). The HLA-DQB1*05 gene showed significantly elevated expression in children with acute lymphoblastic leukemia (ALL) compared to healthy controls in Iranian research [17]. Recent reports have identified additional genetic markers associated with heightened vulnerability to ALL, including the HLA-DRB1*04 allele, which was found in Iranian patients diagnosed with ALL (*p* = 0.027) [18]. A Turkish investigation identified HLA-A*25 and DRB1*04 as potential genetic risk factors for pediatric ALL patients [19]. A separate Turkish study identified elevated frequencies of the DRB1*04 and DRB1*07 alleles in patients categorized as high risk and standard risk, respectively (*p* = 0.009 and *p* = 0.007), indicating that DRB1*04 may predispose individuals to acute lymphoblastic leukemia (ALL). The research indicated that the DRB1*07 allele may be linked to standard risk in ALL patients [20]. A recent study revealed HLA-DRB1*16 as an additional possible genetic risk factor [21]. The HLA-DRB1*11 and HLA-A*32 alleles are particularly noteworthy for their possible predictive importance in juvenile acute lymphoblastic leukemia (ALL) [22].

Conversely, our investigation identified strong protective alleles HLA-B*49, HLA-B*40, and HLA-B*57 for pediatric leukemia (*p* < 0.001, *p* = 0.05, *p* < 0.04, respectively), which was similarly recognized in the U.S. population [8]. A recent Turkish study indicated that HLA-B*55 may serve as a protective factor against both ALL and AML [19]. A recent study found the HLA-DRB1*07 and HLA-DRB1*12 alleles to be protective factors in patients with acute lymphoblastic leukemia (ALL) [21]. Other protective alleles, including HLA-A*26 (*p* = 0.025), HLA-A*33 (*p* = 0.02), and HLA-DRB1*03 (*p* = 0.035), were shown to exist at markedly reduced frequencies in the patient cohort [18]. An Algerian study indicated that in patients from western and southwestern Algeria, HLA-B*27 and HLA-B*58 may be correlated with an elevated risk of acute leukemia [23]. Nonetheless, it is crucial to acknowledge that, due to the sample size, there is no agreement on these relationships, whether affirmative or adverse. Numerous investigations, particularly those involving Indian [12] and Romanian patients, have produced outcomes that contradict our findings [13].

Concerning the association of HLA molecules with BCP-ALL, significantly elevated frequencies have been observed in affected children compared to controls; however, this association is exclusively linked to class II HLA molecules, specifically DRB1*01 (*p* < 0.001) and DQB1*05 (*p* < 0.01). In reviewing our findings, we identified only one study conducted in the United Kingdom, which reported no association between childhood BCP-ALL and variations in the major histocompatibility complex (MHC) [3].

Leukemia is more prevalent in boys than in girls, although the underlying reasons are not well understood. Our investigation revealed no significant gender-based differences in the distribution of HLA-DRB1 alleles. An Egyptian investigation revealed that the HLA-DRB1*04 allele may serve as a female-specific susceptibility factor for pediatric acute lymphoblastic leukemia (ALL) and could affect the age of onset [22]. A further investigation indicated notable correlations with HLA-A*33 and HLA-DRB1*12 alleles in both male and female cohorts [12].

Our work indicates that the correlation identified between HLA class I and II alleles may serve as a potential predisposing factor for the development of juvenile acute lymphoblastic leukemia (ALL). Nevertheless, further comprehensive research is required to ascertain the protective and predisposing functions of particular HLA alleles.

## 4. Materials and Methods

### 4.1. Study Design

We performed a retrospective cross-sectional controlled study involving 70 children diagnosed with acute lymphoblastic leukemia (ALL)—39 with BCP-ALL, 23 with TCP-ALL, and 8 unclassified cases—alongside 136 healthy control children from the southern regions of Morocco, including the Marrakech–Safi (representing 70% of children with ALL and the same proportion in the control group), Guelmim-Oued Noun (12% of the studied population), Beni Mellal-Khenifra (8% of the studied population), Souss-Massa (6% of the studied population), and Draa-Tafilalt (4% of the studied population) governorates. The recruitment period extended from 2014 to 2024. The diagnosis of acute lymphoblastic leukemia (ALL) was determined using Complete Blood Count and peripheral smear findings in conjunction with Bone Marrow Examination. The inclusion criteria were rigorously defined by a detailed examination of the individuals’ medical histories, in conjunction with the pediatric hematology department. Patients and controls were chosen from all patients presented to the HLA laboratory of the University Hospital, either as candidates for hematopoietic stem cell transplantation (HSCT) or as prospective healthy donors. This study excluded children with myeloid leukemia and adult patients.

### 4.2. Ethical Considerations

The patient and control samples used in this investigation were acquired using the standard operations of the HLA laboratory. Sociodemographic and clinical data were retrieved anonymously from the database under the oversight of the laboratory manager. In this instance, ethical approval and informed consent were not required.

### 4.3. Sample Collection and Processing

Peripheral venous blood was obtained using two 5 mL tubes containing Ethylene-diaminetetraacetic Acid (EDTA) as an anticoagulant, in accordance with a meticulously established methodology for HLA typing. Following collection, the samples were placed in designated transportation bags and promptly delivered to the laboratory to preserve sample integrity throughout transit, which is essential to prevent deterioration and ensure the preservation of cellular components required for precise HLA typing.

### 4.4. DNA Extraction

The QIAmp DNA Mini kit (Qiagen, Hilden, Germany) was employed to extract genomic DNA from peripheral blood mononuclear cells (PBMCs) using a multi-step protocol. The initial phase of this procedure involved cell lysis, wherein PBMCs were subjected to a lysis buffer containing chaotropic salts to disrupt cell membranes and release genomic DNA. Subsequently, the lysate was applied to a silica-based column, allowing the genomic DNA to be eluted, as it was specially adhered to the silica membrane. The DNA was purified using a sequence of ethanol-based wash buffers to remove salts, metabolites, and other contaminants. Finally, an elution buffer was employed to meticulously and without harm extract the isolated DNA from the membrane.

The resultant DNA was quantitatively and qualitatively evaluated using a NanoDropTM 2000/2000c Spectrophotometer (Thermo Scientific™, Waltham, MA, USA), which determined DNA concentration and purity, essential for subsequent molecular investigations.

### 4.5. HLA Typing

HLA class I (A and B loci) and class II (DRB1 and DQB1 loci) typing was performed utilizing two separate polymerase chain reaction (PCR) platforms, employing sequence-specific oligonucleotide (SSO) PCR, supplied by Immucor™ (LIFECODES^®^ HLA-SSO Typing, Peachtree Corners, GA, USA) and Onelambda (Thermo Fisher Scientific, LabType™, Waltham, MA, USA). This method entails PCR amplification succeeded by the hybridization of the amplified products to beads coated with oligonucleotide probes specific to known HLA sequences.

The Luminex technology is employed to assess hybridization patterns and detect fluorescent signals from the microspheres. The HLA A, B, DRB1, and DQB1 loci and specificities in the sample can be seen using the color coding of each microsphere, corresponding to a specific HLA probe. The fluorescence intensity also indicates the extent of hybridization.

The analysis of the supplied PCR typing data was conducted using Fusion 4.4^®^ (One Lambda) version 4.3.1 or MATCH IT!^®^ (Immucor) software version 1.4, incorporating fluorescence intensity data via Luminex xMAP technology. These software systems employ a database of established allele sequences to correlate with the observed probe fluorescence patterns, delivering an extensive profiling of HLA class I and class II loci and specificities for patients and controls.

### 4.6. Statistical Analysis

Data analysis was conducted using IBM SPSS Statistics version 29.0 (IBM, Armonk, NY, USA), and the χ^2^ test was employed to determine the statistical significance of variations in HLA allele group frequencies between patients and controls. We utilized Jamovi v.1.2 (The Jamovi Project, Sydney, Australia) to generate the graphs for each significant allelic group. Results were deemed statistically significant when the *p*-value was less than or equal to 0.05.

## 5. Conclusions

The findings of our study indicate that HLA-A*63, -B*14, -DRB1*01, and DQB1*05 may serve as potential predisposing immunogenetic variables for the development of pediatric acute lymphoblastic leukemia (ALL). Additionally, our research shows that the frequencies of HLA-DRB1*01 and DQB1*05 were elevated in pediatric ALL B compared to the control group. It would be beneficial to expand these findings to a bigger cohort study to validate the results and contribute substantial new elements to the existing research.

## Data Availability

The data used in this study are not publicly available due to privacy restrictions. Access to the data is restricted in accordance with the ethical guidelines and regulations governing the protection of participant confidentiality and privacy. However, researchers interested in replicating or verifying the findings presented in this study may request access to the data through the appropriate institutional review board. Requests for data access will be considered on a case-by case basis, subject to approval by the relevant authorities and compliance with applicable privacy regulations. For inquiries regarding data access, please contact Pr. Brahim ADMOU/Clinical research Center/Mohammed VI University Hospital Center at br.admou@uca.ac.ma.

## Abbreviations

The following abbreviations are used in this manuscript:

ALL	Acute lymphoid leukemia
SSOs	Sequence-specific oligonucleotides
SSPs	Sequence-specific primers
PCR	Polymerase chain reaction
HLA	Human leukocyte antigen
BCP-ALL	B cell precursor acute lymphoblastic leukemia
TCP-ALL	T-cell precursor acute lymphoblastic leukemia
MHC	Major histocompatibility complex
IARC	International Agency for Research on cancer
